# Cerebral Vasospasm in Patients With Severe Traumatic Brain Injury

**DOI:** 10.1186/2197-425X-3-S1-A490

**Published:** 2015-10-01

**Authors:** A Karpunin, S Petrikov, L Hamidova, V Krylov

**Affiliations:** Regional Hospital of the Ministry of Healthcare of Russian Federation, Ryazan, Russian Federation; Institute of Emergency Care named N.V. Sklifosofskiy, Moscow, Russian Federation, Moscow, Russian Federation

## Introduction

Cerebral vasospasm (CV) occurs in 18-70% of patients with severe traumatic brain injury (sTBI) and is one of the main reasons for the development of secondary ischemic brain damage and a risk factor for adverse neurodevelopmental outcome.

## Objectives

Determine the frequency and timing of cerebral vasospasm (CV) development in patients with severe traumatic brain injury (sTBI) and assess its impact on patient outcomes.

## Methods

The study included 43 patients with isolated and combined sTBI and Glasgow Coma Scale on admission 8 or less (age 32.4 ± 10.8 years, men/ women - 36/7). Severe brain contusion was diagnosed in all patients. Emergency surgery was required in 20 patients. All patients received decompressive craniotomy with intracranial hematoma or contusion foci removal. Transcranial duplex investigation was performed every day from 2 till 11 day after admission. We measure blood flow velocity, Lindegaard index, pulsatility index, overshoot ratio. Cerebral vasospasm was diagnosed in case of systolic blood flow velocity (Vs) in the middle cerebral artery 120 cm/sec or more with Lindegaard index 3 or more.

## Results

CV was detected in 33 (77%) patients. in 16 patients (48%) we diagnosed moderate (Vs 120-200 cm / sec) and in 17 (52%) - severe (Vs > 200 cm / sec) vasospasm. Moderate CV developed at the 4-5 day after injury and was unilateral in 4(25%) and bilateral in 12 (75%) patients. Severe CV developed within the first 3 days after injury and was unilateral in 2(12%) and bilateral in 15 (88%) patients. the duration of the CA exceeded 11 days in all patients. Cerebral vasospasm development was associated with increase in bad outcomes, evaluated by Glasgow Outcome Score (1 - Dead, 2 - Vegetative state, 3 - Severe neurological impairment, 4- moderate neurological impairment, 5 - Full recovery). GOS 1-3 was noted in 30% (n = 3) patients without signs of CV, in 31% (n = 5) patients with moderate CV and in 82% (n = 14) patients with severe CV.
